# 78046 The Kansas City Quality & Value Innovation Consortium (KC QVIC): Leveraging Team Science, Translational Research and Training to Improve the Value of Healthcare in the Community

**DOI:** 10.1017/cts.2021.686

**Published:** 2021-03-30

**Authors:** Stacy Farr, Jon Spertus, James Stowe, Holly Hagle, Jacque Sawyer

**Affiliations:** 1 Saint Luke’s Health System and University of Missouri-Kansas City; 2 Mid America Regional Council (MARC); 3 University of Missouri-Kansas City

## Abstract

ABSTRACT IMPACT: Implementing a team science approach with broad engagement from academic researchers, healthcare payers, providers, patients, and community-based organizations is complex, yet critical to implementing evidence into real world settings. OBJECTIVES/GOALS: 1. Participants will be able to deploy novel strategies for creating and training a regional multi-stakeholder consortium to improve the quality and value of healthcare.

2. Participants will be to examine ways in which team science provides holistic sustainable strategies to improve care and outcomes in real-world settings. METHODS/STUDY POPULATION: The Quality & Value Innovation Consortium (QVIC) has created a network of hospitals and other stakeholders (providers, payers, purchasers, patients, community-based organizations, and researchers) to collaborate and innovate on healthcare delivery. This initiative began with a team of a physician researcher, a health services researcher, and a nurse researcher first identifying healthcare systems’ priorities through individual meetings with leadership from 14 regional hospitals. Concurrently, meetings were held with other stakeholders. These interviews identified 32 key quality improvement topics.

Focus groups and surveys reduced these to 11 topics that were then selected for community forums. Through a mixed methods approach, two priority topics were selected for regional implementation. RESULTS/ANTICIPATED RESULTS: The QVIC meetings have prioritized two topics and highlighted novel information sharing across entities, and strategies to address the social determinants of health. The QVIC efforts have been recognized as a community asset for helping build collaboration and partnerships among diverse stakeholders. Ultimately, two regional initiatives, opioid management, and transitions in heart failure care were selected for implementation. Both of these initiatives aim to reduce readmissions by addressing social determinants of health. Implementation strategies and evaluation metrics are being customized for pragmatic integration within each system, utilizing a collaborative team science approach. DISCUSSION/SIGNIFICANCE OF FINDINGS: While the entire country is grappling with the challenge of improving the quality of care, while lowering its costs, Kansas City has modeled a unique culture and strategy for achieving this goal, important for learning health systems and communities.

